# Does p-coumaric acid improve cardiac injury following LPS-induced lung inflammation through miRNA-146a activity?

**Published:** 2020

**Authors:** Maryam Kheiry, Mahin Dianat, Mohammad Badavi, Seyyed Ali Mard, Vahid Bayati

**Affiliations:** 1 *Department of Physiology, Physiology Research Center, Faculty of Medicine, Ahvaz Jundishapur University of Medical Sciences, Ahvaz, Iran*; 2 *Cellular and Molecular Research Center, Faculty of Medicine, Ahvaz Jundishapur University of Medical Sciences, Ahvaz, Iran*

**Keywords:** LPS, p-coumaric acid, Acute lung injury, miRNA 146a, Cardiac inflammation

## Abstract

**Objective:**

In cardiovascular diseases, inflammatory response plays an important role and affects heart function. As a flavonoid compound, p-coumaric acid (pCA), commonly exists in many fruits and vegetables and has a therapeutic effect on inflammatory diseases due to its anti-inflammatory properties. The purpose of the present study was to investigate pCA anti-inflammatory effect and the miRNAs (miRs) signaling pathway involved in cardiac inflammation following lipopolysaccharide-induced acute lung injury (ALI).

**Materials and Methods:**

Thirty-two Sprague-Dawley male rats were divided into 4 groups: control (received saline for 10 days, i.p.), LPS (received saline for 10 days+5 mg/kg LPS on day 8, intratracheally), pCA (received pCA 100 mg/kg for 10 days, ip), and LPS+pCA (received LPS+pCA). The level of IL-1β, IL-18 in heart tissue and IL-1β in bronchoalveolar lavage fluid (BALF) was determined by ELISA kits. Also the level of lactate dehydrogenase (LDH) in heart tissue and myeloperoxidase (MPO) in lung tissue were measured, and pCA effect on miR- 146a in heart tissue was analyzed.

**Results:**

Data showed that 100 mg/kg of pCA significantly suppressed LDH activity (p<0.05), IL-18 (p<0.05) and IL-1β (p<0.01) level in heart tissue. Also, in BAL, IL-1β and MPO levels were significantly reduced (p<0.001). Finally, pCA modulated activation of miR-146a (p<0.05) in LPS -induced cardiac injury.

**Conclusion:**

These findings indicated that LPS causes cardiac dysfunction and pre-treatment with pCA, as an anti-inflammatory agent, improved cardiac inflammation through modulation of miR-146a, and reducing cytokines and LDH activity.

## Introduction

Inflammation is the first line of defense against a variety of factors and is properly regulated by different biological responses (Henson, 2005 ▶). Acute lung injury (ALI)-induced by lipopolysaccharide (LPS), causes systemic inflammation which can affect distant organs lead to endothelial dysfunction (Rodríguez-González et al., 2014 ▶). In the pathophysiology of ALI and cardiovascular diseases, dysregulated inflammation reflected by increasing levels of proinflammatory cytokines and abnormal immune responses, has a pivotal role (Rocha and Libby, 2009 ▶). Microbial components such as LPS which are known as inflammatory stimuli, trigger expression of inflammatory mediators, inflammatory microRNAs (miRs), pro-inflammatory cytokines such as tumor necrosis factor (TNF-α) (Baltimore et al., 2008 ▶), and miRNAs as single-stranded noncoding RNAs, and modification of gene expression at post-transcription level (Kim, 2005 ▶) and can act as positive or negative regulators in inflammatory condition (Baltimore et al., 2008 ▶). One of the miRNAs that is induced by inflammatory stimuli is miR-146a, which has a vital function in duration and severity of an immune response. Under inflammatory conditions and in case of suppressed cytokine production, miR-146a acts as a negative regulator (Saba et al., 2014 ▶).

LPS leads to systemic infection which is associated with increased permeability of the systemic capillaries and it can even result in left ventricular (LV) dysfunction, alteration of systemic vascular resistance, disturbance in ejection fraction, and ventricular dilation and increased cardiac output (Rudiger and Singer, 2007 ▶). 

 Herbal products are rich in phenolic compounds and they are frequently used by humans and animals (Bursal et al., 2013 ▶; Topal and GÜLÇİN, 2014 ▶). Several studies found flavonoids and phenolic acids in natural antioxidants (Çakmakçı et al., 2015 ▶; Arabaci et al., 2014 ▶). P-coumaric acid (pCA) that is found in many plant products including potatoes, beans, tomatoes and tea (Gülçin et al., 2010 ▶), and vegetables (Innocenti et al., 2010 ▶), is a phenolic compound (Gülçin et al., 2010 ▶). pCA was reported to decrease stomach cancer and heart disease (Tohma and Gulçin, 2010 ▶; Yoon et al., 2014 ▶).

The effect of ALI on the heart is less clear, and antioxidant therapy can be effective. Therefore, in this research, the effect of pCA on LPS-caused cardiac injury was investigated with respect to LDH activity, proinflammatory markers levels and miRNA 146a activity.

## Materials and Methods

 LPS (*Escherichia coli* LPS, 055:B5), and p-coumaric acid (pCA) were purchased from Sigma (Sigma-Aldrich, USA), and xylazine 2% and ketamine HCl 10% (Alfasan Co. Netherlands), and ELISA kits were obtained from Diaclone, France.

Male Sprague-Dawley rats (weighing 200–250 g) were purchased from the Animal House of Ahvaz Jundishapur University of Medical Sciences. The rats were maintained in an animal house with constant temperature and central air conditioning. In this experiment, 32 rats were randomly divided into the following 4 groups: (1) control rats which received saline for ten day; (2) LPS rats which received saline for ten day+LPS (5 mg/kg, intratracheally injection) on the 8^th^ day (Deng et al., 2015); (3) pCA rats received pCA (100 mg/kg) for ten day (Pragasam et al., 2013); and (4) rats that received LPS (5 mg/kg)+pCA (100 mg/kg). In the LPS+pCA group, rats were pre-treated with 100 mg/kg pCA for 10 days and on the 8^th^ day, LPS was instilled into the airways. The rats were sacrificed 72 hr after LPS injection.

**LPS instillation**

 The model of ALI was induced by injection of 5 mg/kg of *E. coli* LPS into the airways (intratracheally) after anesthetizing rats with xylazine and ketamine (ip). Control animals received normal saline through the same route (Deng et al., 2015 ▶).

**Bronchoalveolar lavage procedure**

 In the end of the protocol, thoracic cavities of rats were opened, and each lung sample was lavaged with PBS for three times. The BAL fluid (BALF) was centrifuged and the supernatant was stored at -80°C for future examinations.

**IL -1β production in BALF**

 IL-1β levels in BALF supernatant were analyzed using an ELISA kit according to the manufacturer’s instructions (Diaclone).

**MPO activity in lung tissue**

In the lung, accumulation of neutrophils was determined in terms of myeloperoxidase (MPO) activity. After freezing the lung tissue samples, in cool normal saline, homogeneity was performed, and homogenates were prepared according to the manufacturer’s instructions. MPO activity was determined spectrophotometrically at 460 nm.

**Measurement of LDH activity in heart tissue**

 LDH activity was spectrophotometrically determined in supernatant from heart tissue homogenates, according to the manufacturer’s instructions by using LDH determination kit (Pars Azmun, Tehran, Iran) to evaluate the cardiac injury.

**IL -1β and IL-18 production in heart tissue**

 Proinflammatory cytokines levels (IL-1β and IL-18) in heart tissue were measured to confirm lung inflammation induction by LPS in all groups. ELISA kits were employed according to the manufacturer’s instructions (Diaclone).

**MicroRNAs extraction, cDNA synthesis and quantitative real-time PCR**

 Frozen heart tissue samples were used in all groups and total miRNAs was extracted using miRNeasy Plus Mini kit (QIAGEN, GmbH, Germany). The purity and concentration of RNA were determined spectrophotometrically (wavelengths 260 and 280 nm, NanodropThermo Scientific S.N:D015). Synthesis of cDNA was done using miScript II RT kit (QIAGEN, GmbH, Germany) 

 By quantitative real-time polymerase chain reaction (qRT-PCR), the expression level of miRNA was determined using a Light Cycler_ 96 Real-Time PCR System [Roche Diagnostics, Indianapolis, IN, USA), miR-146a (MS00000441; QIAGEN], Universal Primer (MS0003374; (QIAGEN)], The levels of miRNAs expression were normalized against RNU6 (as an internal control) and the fold change was calculated using the 2^−ΔΔCt ^method (Akbari et al., 2017 ▶).

**Statistical analysis**

 Data are presented as mean±SEM and data comparisons were made by Student’s t-test or one-way analysis of variance followed by Tukey-Kramer multiple comparisons test. Differences were considered significant if p<0.05.

## Results

**IL-1β activity in BALF in ALI**

**Figure 1:**
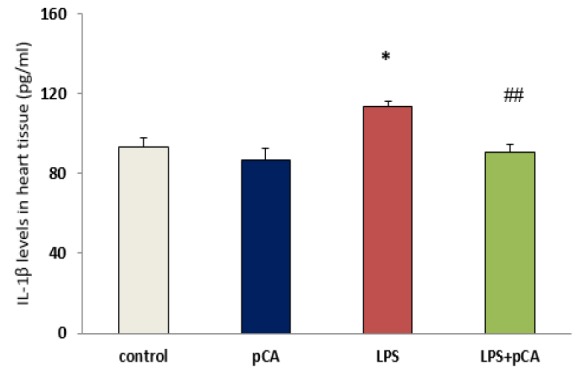
PCA pre-treatment effects on cardiac injury following LPS-induced lung inflammation. IL-1β level in BALF of ALI rat was measured by ELISA. Values are expressed as mean±SEM (n=8). ***p<0.001 shows significant differences as compared to control group;

To confirm induction of acute lung injury by LPS, the level of IL-1β in BALF was determined. Significant increases in IL-1β were observed in ALI rats (p<0.001). Pre-treatment with pCA in LPS group suppressed IL-1β production compared to LPS rats (p<0.001) (Figure 1).

**MPO activity in lung tissues in ALI**

**Figure 2:**
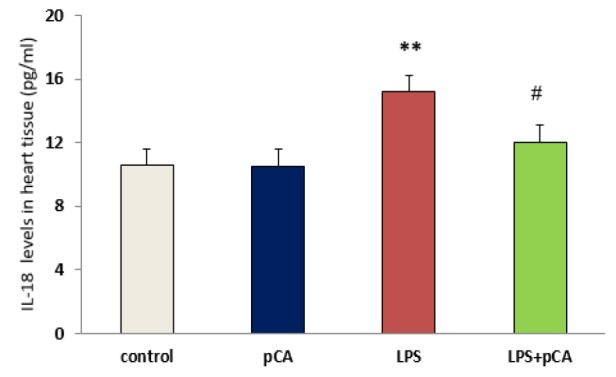
PCA pre-treatment effects on cardiac injury following LPS-induced lung inflammation. MPO activity in lung tissue of ALI rat was measured. Values are expressed as mean±SEM (n=8). ***p<0.001 shows significant differences as compared to control group; ###p<0.001 shows significant differences as compared to LPS group

 MPO are important cytokines used as a marker of lung inflammation status. Levels of MPO activity in lung tissue were determined. Significant increases in MPO activity (p<0.001) were observed in ALI rats. However, MPO activity significantly decreased by pre-treatment with pCA in ALI rats compared to ALI alone (p<0.001) (Figure 2).

###p<0.001 shows significant differences as compared to LPS group.

LPS=Lipoplysaccharide, pCA=P-Coumaric acid.

LPS=Lipoplysaccharide, pCA=p-coumaric acid.

**Cytokines levels in heart tissues**

**Figure 3:**
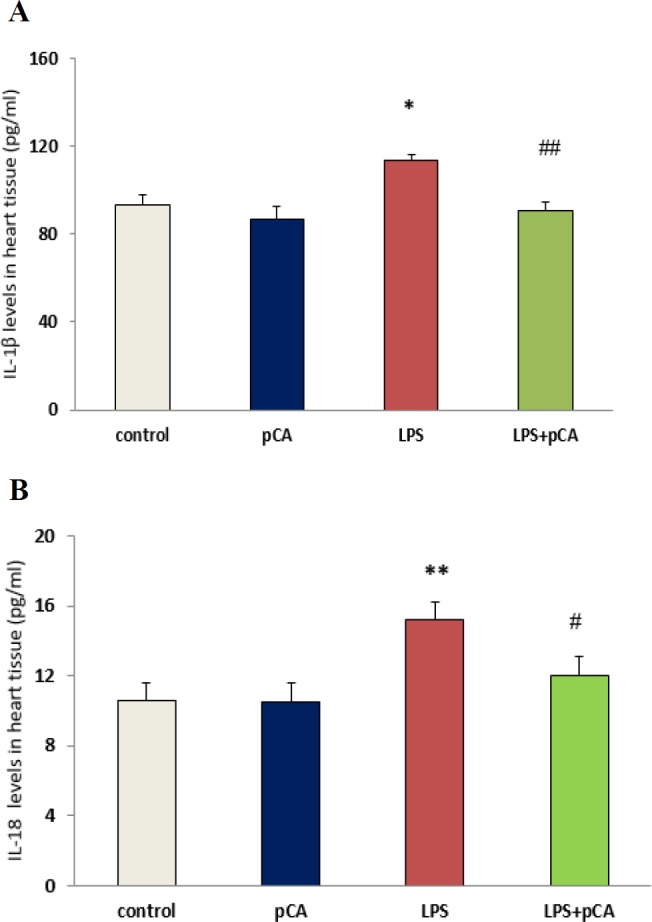
PCA pre-treatment effects on cardiac injury following LPS-induced lung inflammation. IL-1β (A) and IL-18 (B) levels in heart tissue of ALI rat was measured by ELISA. Values are expressed as mean±SEM (n=8). *p<0.05 and ** p<0.01 shows significant differences as compared to control group; #p<0.05 and ##p<0.01shows significant differences as compared to LPS group

 IL-18 and IL-1β levels are regarded as inflammation indices. Significant increases in IL-1β (p<0.05) and IL-18 (p<0.01) activity were observed in ALI rat. However, IL-1β and IL-18 activity was significantly decreased by pre-treatment with pCA in LPS group compared to LPS alone (p<0.01 and p<0.05, respectively) (Figure 3).

**LDH activity in heart tissue**

**Figure 4:**
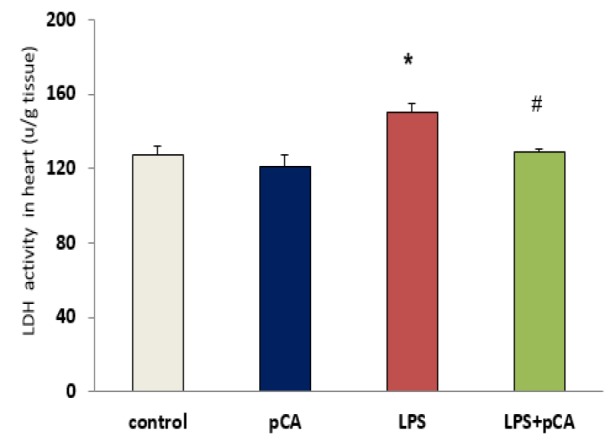
PCA pre-treatment effects on cardiac injury following LPS-induced lung inflammation. The level of LDH activity in heart tissue of ALI rat. Values are expressed as mean±SEM (n=8). *p<0.05 shows significant differences as compared to control group; #p<0.05 shows significant differences as compared to LPS group

 As shown in Figure 4, significant increases in LDH (p<0.05) activity were observed in heart tissue of ALI rat. However, LDH activity significantly decreased by pre-treatment with pCA in LPS group compared to LPS alone (p<0.05).

LPS=Lipoplysaccharide, pCA=p-coumaric acid.

**qRT-PCR for miRNA146a gene expression in heart tissue**

**Figure 5:**
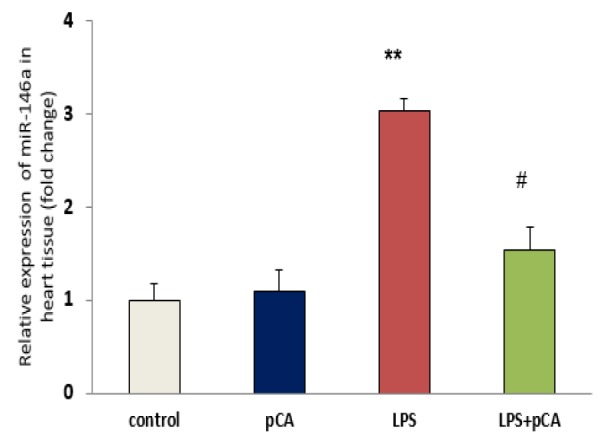
PCA pre-treatment effects on cardiac injury following LPS-induced lung inflammation. The relative expression of miR-146a in heart tissue of ALI rat was measured by qRT-PCR. Values are expressed as mean±SEM (n=8). **p<0.01 shows significant differences as compared to control group; #p<0.05 shows significant differences as compared to LPS group

 According to qRT-PCR data, significant increases in miR-146a level were observed in heart tissue of ALI rat (p<0.01). However, these levels significantly decreased (p< 0.05) by pre-treatment with pCA in LPS group compared to LPS alone (Figure 5).

LPS=Lipoplysaccharide, pCA=p-coumaric acid.

LPS=Lipoplysaccharide, pCA=p-coumaric acid.

**Figure 6:**
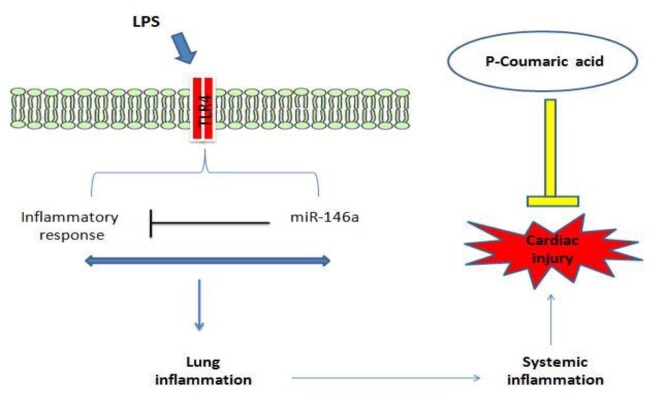
Schematic diagram showing the mechanism of LPS induced- cardiac injury and protective role of p-coumaric acid

## Discussion

To confirm the induction of acute lung inflammation by LPS, IL-1β level in the BALF and MPO activity in lung tissue of LPS rats, were measured. According to our results, significant increases in IL-1β and MPO activity were observed following intratracheal administration of LPS. 

 However, these results indicated high levels of pro-inflammatory cytokine such as IL-18 and IL-1β, in heart tissue following intratracheal administration of LPS in rats with ALI. Local inflammatory reaction can be induced by LPS (as one of the most important causes of the lung inflammation), which has a critical role in ALI and acute respiratory distress syndrome (ARDS) diseases (Abraham, 2003 ▶). However, in different animal models, cytokine levels can be reduced in order to attenuate lung injury (Nadeau-Vallée et al., 2017 ▶; Wu et al., 2013 ▶). LPS administration was reported to lead to NF-κB activation in most studies (Lee et al., 2015 ▶) which in turn, causes myocardial dysfunction by increasing the expression of myocardial cytokines TNF-α and IL-1β (Baumgarten et al., 2001 ▶).

Indeed, LPS induced acute lung injury (ALI) and potentially by releasing inflammatory mediators into the circulation, probably contributes to a failure in multiple organs such as the heart, and affects their function.

 LDH activity as a marker of heart injury was determined in heart tissue. Our results demonstrated that 72 hr after LPS treatment, there was an increase in LDH activity. We tested the effect of pCA in ALI rats to assess pCA anti-inflammatory effects. We found that pre-treatment with pCA effectively suppressed IL-1β, IL-18 and LDH production in heart tissue of LPS rats. Therefore, one of the important therapeutic strategies for reducing tissue damage and maintaining immune homeostasis can be the reduction of IL-1β, IL-18 and LDH levels following exposure to pathogens.

Disturbances in cellular redox balance due to increased reactive oxygen species (ROS) production or impaired antioxidant defense (an imbalance between oxidants and antioxidants) ultimately lead to oxidative changes in biocompatible macromolecules including lipids, proteins, and DNA (Rahman and Adcock, 2006 ▶). pCA as an antioxidant and anti-inflammatory agent, is able to neutralize the effects of free radicals in heart tissue and prevent inflammation in the heart. 

 Different studies indicated that pCA, in addition to its antidepressant properties (Lee et al., 2018 ▶), has various effects such as anti-oxidant, anti-angiogenic, anti-UV damage, and anti-platelet (Yoon et al., 2014 ▶).

Many studies showed that miRNAs (miRs) have an important role in regulating human diseases. Several reports showed that miRs play a critical function in tumor genesis (Buechner et al., 2011 ▶), cardiomyopathy (Ono et al., 2011 ▶) and chronic inflammatory diseases (Oglesby et al., 2010 ▶).

 To investigate signaling pathway through which pCA acts in ALI rats, we examined the expression of miR-146a in heart tissue in all groups. Our data showed increased expression of miR-146a in heart tissue 72 hr after induction of cardiac injury by LPS, which is in agreement with previous studies (Ruggiero et al., 2009 ▶; Arango et al., 2015 ▶). Among the inflammatory miRs, miR-146a is a regulating miR which acts in a negative feedback loop to reduce the severity of the tissue damage caused by inflammation. 

 It was observed that the exposure of human monocytes and RAW264.7 macrophage cells to LPS, leads to increases in inflammatory response that result in activation of miR-146a (He et al., 2014 ▶). 

 Our results showed that pre-treatment with pCA effectively modulated the expression of miR-146a in heart tissue in ALI rats. pCA was able to prevent inflammation induced by LPS (Figure 6), which could be one of the reasons for the decline in miR-146a expression. Tsai et al. (2012) ▶ showed that *Calophyllum inophyllum* L. extract, an anti-inflammatory agent inhibited the increase in miR-146a expression in RAW 264.6 cells following LPS exposure (Tsai et al., 2012 ▶) which is in line with our findings that pCA as a flavonoid has an anti-inflammatory effects in LPS-induced lung inflammation.

 In summary, the results of this research indicated that pCA could attenuate cardiac injury in a LPS-induced ALI model; this effect was at least in part, caused through inhibition of an inflammatory miRNA, and down-regulation of inflammation mediators and LDH activity as a cardiac injury marker.
